# Cottage Hospital Construction

**Published:** 1893-11-11

**Authors:** 


### PRACTICAL POINTS.

[Questions are invited for this column on any point in the administration of hospitals and asylums which may prove of difficulty or interest to our readers. All communications should be marked "Practical Points," addressed to the Editor of THE HOSPITAL, 428, Strand, W.C.]

### COTTAGE HOSPITAL CONSTRUCTION.

*Cottage Hospital Walls.—What is the best way of treating the walls of a cottage hospital built four years ago? They are at present distempered. Is paint best? or is there any other substance not too expensive that can be washed? Not tiles; these would be considered too expensive. The hospital contains small isolation ward used for scarlet fevers.*

The best way to treat the walls of a cottage hospital such as you describe is to select papers of an attractive pattern, have them properly sized, and then varnished carefully with the best white copal varnish. The paper should be varnished once; then left for a year, so as to get thoroughly hard and dry; then sponged and varnished again. It will thus stand for ten or fifteen years if properly done. Not only is this plan hygienically perfect, but it will enable you to make each ward bright and pretty in its own way, as you can thus secure variety of colour both in paper and the paint used for the woodwork. It is best to procure the paper direct from the manufacturer, so as to secure that the sizing should be properly done. The cost will not exceed that of the best oil paint, and the result will be found infinitely more satisfactory.

